# Modified Thermocouple Sensor and External Reference Junction Enhance Accuracy in Indoor Air Temperature Measurements

**DOI:** 10.3390/s21196577

**Published:** 2021-10-01

**Authors:** Hans Lundström, Magnus Mattsson

**Affiliations:** Department of Building Engineering, Energy Systems and Sustainability Science, University of Gävle, SE-801 76 Gävle, Sweden; magnus.mattsson@hig.se

**Keywords:** air temperature measurement, high accuracy, temperature sensor, thermocouple, external cold-junction compensation, radiation, induced errors

## Abstract

Indoor air temperature belongs to the most important climatic variables in indoor climate research, affecting thermal comfort, energy balance, and air movement in buildings. This paper focuses on measurement errors when using thermocouples in indoor temperature measurements, with special attention on measurements of air temperature. We briefly discuss errors in thermocouple measurements, noting that, for temperatures restricted to indoor temperature ranges, a thermocouple Type T performs much better than stated in “standards”. When thermocouples are described in the literature, industrial applications are primarily considered, involving temperatures up to several hundred degrees and with moderate demands on accuracy. In indoor applications, the temperature difference between the measuring and the reference junction is often only a few degrees. Thus, the error contribution from the thermocouple itself is almost immeasurable, while the dominant error source is in the internal reference temperature compensation in the measuring instrument. It was shown that using an external reference junction can decrease the measurement error substantially (i.e., down to a few hundredths of a degree) in room temperature measurements. One example of how such a device may be assembled is provided. A special application of room temperature measurements involves measuring indoor air temperature. Here, errors, due to radiation influence on the sensor from surrounding surfaces, were surprisingly high. The means to estimate the radiative influence on typical thermocouples are presented, along with suggestions for modification of thermocouple sensors to lower the radiation impact and thereby improve the measurement accuracy.

## 1. Introduction

### 1.1. Background

Temperature is perhaps the most measured climatic variable in indoor climate research. Indoor air temperature is an essential parameter for the thermal comfort and energy balance in a building [1,2]. There is a need for increased accuracy in indoor climate measurements; moreover, due to the increasing use of computerized simulation methods, there is a need for high-quality verification data, to develop reliable software models [3,4,5]. Thermocouples are the most commonly used sensors for indoor temperature measurements, at least within the research. They are easy to assemble, can be made very small, and they do not suffer from self-heating, which is beneficial in a low-velocity environment.

However, by far, the most prominent application area for thermocouples involves measuring high temperatures in harsh industrial environments [6]. Thermocouples can be made very robust, and can withstand high temperatures (essentially up to the melting point of the thermocouple metals), and are frequently described in the literature as crude devices with low accuracy. Accordingly, commercially available instrumentation for thermocouples is mainly optimized for industrial use and may not be the best choice for indoor temperature measurements [7]. As we discuss later, the measurement errors for room temperature measurements are dominated by errors in the reference junction. This means that accuracy cannot be enhanced by individual calibration of the thermocouples (a common proposal for solving the problem).

Accurate measurements of indoor air temperatures place unique demands on sensors. However, simple steps to modify sensors and instruments may increase measuring accuracy considerably. In this paper, we highlight the applicability of thermocouples to measurements in the room temperature range, and discuss the adaption of commercial data loggers for these kinds of applications.

### 1.2. Outline of the Paper

In Section 2, the basic principle behind the thermocouple is outlined, and in Section 3, common error sources are discussed. Section 4 focuses on issues special to thermocouples in indoor temperature applications. Assembling an external cold junction compensation device to increase the measurement accuracy is demonstrated and evaluated in Section 5. Section 6 is devoted to air temperature measurements. The influence from radiation on a thermocouple sensor is highlighted, and solutions for lowering errors due to radiation impact are provided. Section 7 summarizes the work.

## 2. Basic Concept of the Thermocouple

From a practical point of view, the thermocouple is one of the simplest of all sensors; it consists of two wires of dissimilar metals joined at the measurement point. However, the theory behind the thermocouple is rather subtle, and for effective use of the sensor, basic knowledge of the governing principles behind the thermocouple is necessary. Mainly, soft soldered bare junction thermocouples are used in indoor climate applications. Typically, two thermocouple wires are tied together at the measuring end, and the reference junction of the thermocouple is created where the wires terminate into the input connector of the measuring instrument. A thermocouple generates a voltage related to the temperature difference between the measuring and the reference junction of the thermocouple (somewhat improperly often named hot and cold junctions). A simple explanation of the principle behind the thermocouple is that heat increases the vibration of the atoms in the metal and pushes free electrons to the colder end of a metal wire, which leaves the hot end positively charged relative to the colder end and, thus, a small voltage is developed over the wire. This phenomenon is known as the Seebeck effect, after the German physicist Thomas Seebeck who discovered the principle of the thermocouple in 1822. The generated voltage is unique for each metal. When two wires of different metals are placed in the same temperature gradient and connected in series, a net voltage is produced, which is related to the temperature gradient. There is no general equation for the absolute thermoelectric power of a metal, and each thermocouple type has its own characteristic temperature-to-voltage curve. The curve’s shape depends on the metals or alloys used, their purity, their homogeneity, and their crystal structure. Standardized thermocouples are made from metal or alloy pairs, developed explicitly for use as thermocouples, and are identified by a letter symbol. There are international standards that specify the amount by which standard thermocouple sensors may deviate from ideal behavior. For indoor temperature measurements, copper–constantan thermocouples are mostly used (also named “Type T”). Copper–constantan thermocouples feature low cost, high output voltages (approximately 40 µV/K), and are easy to assemble by soft soldering. (“Constantan” is originally a trademark of Wilbur B. Driver Co, but has become a generic name for an alloy of 54% Cu, 44% Ni, and 1% Mn.)

Contrary to popular definitions of thermocouples, the voltage is actually generated by the temperature gradient along the wires and not at the junctions [8]. Therefore, the quality of the wire material must be maintained over the whole wire length, especially where temperature gradients exist. However, a third metal may be introduced into a thermocouple circuit and have no impact, provided that both ends of this third wire are at the same temperature. This means that the thermocouple measurement junction may be soldered, brazed, or welded without affecting the thermocouple’s calibration, as long as there is no net temperature gradient along with the third metal in the junction.

Since the thermocouple is a differential device rather than an absolute temperature measurement device, the reference junction temperature must be known to get an accurate absolute temperature reading. The reference junction temperature is measured with another temperature-sensitive device, typically a semiconductor sensor, thermistor, or platinum resistance temperature detector. The thermocouple voltage reading combined with the reference junction temperature gives the absolute temperature at the measuring junction. This process is known as “reference junction compensation” (cold junction compensation). It is essential that the reference junction be read as accurately as possible with a sensor kept at the same temperature as the reference junction.

For a more comprehensive description of thermocouples, see, e.g., Childs et al. [9], Kerlin and Johnson [10]. A short historical background of the development of the thermocouple is presented in the preface of Standard ISA-MC96.1-1982 [11].

## 3. Errors in Thermocouple Measurements

Thermocouple accuracy is a complex subject due to a number of factors. Generally, errors in temperature measurements are of three categories: (1) *Sensor errors*; (2) *Instrument errors*; and (3) *Method errors*.

*Sensor errors* are errors in the thermocouple itself and are mainly due to impurities in the metals and inhomogeneities in the wires. Impurities in the composition of the wire metals affect the shape of the temperature–voltage curve and have the character of a “gain error” and, as such, vary with the temperature difference between the junctions.

Inhomogeneities may generate additional voltage, which appears as an offset error. The thermal voltage developed by a thermocouple made from homogeneous wires will be a function of the temperature difference between the measuring and the reference junctions only, because voltages generated from gradients due to any hot or cold regions along the wires will cancel. If, however, the thermocouple wires are not homogeneous and if inhomogeneities are present in a part where a temperature gradient exists, an extraneous voltage will be developed. The presence of inhomogeneities will lead to the measured voltage being characteristic not only of the temperature difference between the junctions but also of the position of the inhomogeneities in the temperature gradient. Inhomogeneities, which are due to lattice deformations, are small for a new wire, but at high temperatures, above a couple of hundreds of degrees, inhomogeneities tend to increase with age. Moreover, cold work on the wires, such as sharp bending, may cause lattice deformations and, thus, increase inhomogeneity. Hence, this type of error, which appears as an offset error, is affected by the environment and the handling of the thermocouple, and cannot be removed by calibration because moving the thermocouple to a calibration rig will change the environment that partly causes the errors. At temperatures above 200 °C, inhomogeneities may be the limiting factors for the measurement accuracy [6] and for high precision calibration of a thermocouple above 200 °C, it is, therefore, necessary to test for inhomogeneity.

Thermocouples are produced in two tolerance classes, Class1 and Class2, also referred to as premium and standard grades. In the Standard IEC 60584-1: 2013 [12], the tolerance for a thermocouple Type T, standard grade, operating in the range 0–350 °C, is specified as ±1.0 K or ±0.75% of a measured value expressed in °C, while a premium grade Type T is specified as ±0.5 K or ±0.4%. The specified tolerances are for a new wire and within the given temperature range. The values given in the Standard are not intended to be used for the calculation of measurement errors, but rather indicate the tolerances to which the producers expect to control the quality of their products.

The offset values refer to inhomogeneity errors. As most applications for thermocouples include temperatures above 200 °C, where varying inhomogeneity may play a role, it is a practice to include in thermocouple calibrations an uncertainty component for inhomogeneity based on a widely accepted rule-of-thumb [13]. However, the given values do not reflect a realistic situation for room temperature measurements, and we must state that it seems that the IEC Standard is mainly established with industrial applications in mind. (“Industrial” here refers to environments with temperatures well above the room temperature range, combined with possible temperature gradients along the thermocouple wires). In principle and in practice, a thermocouple can perform significantly better than the Standard indicates if it is used well below its nominal upper temperature limit, is not affected by cold working, and is protected from large gradients along the thermocouple wires. Moreover, modern manufacturing process controls in the fabrication of the thermocouple wire have increased the quality of metals and alloys, allowing for a tighter accuracy specification.

**Figure 1:**
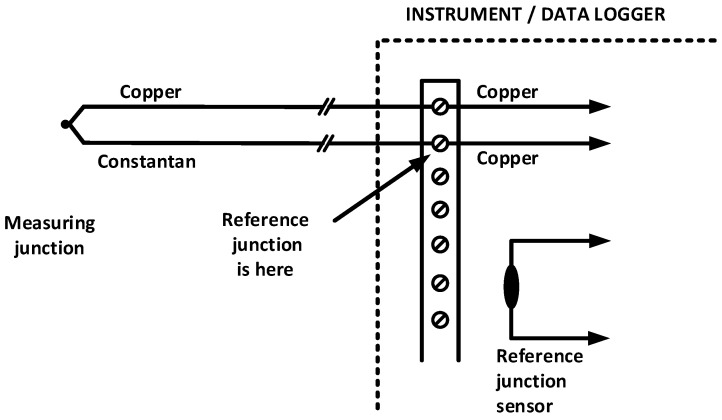
A standard connection of a Type T thermocouple to an instrument. Note the reference junction at the screw terminal.

*Instrument errors* are almost always strongly dominated by errors in the reference junction compensation [7]. Any error in the reading of the reference junction temperature will show up directly in the final thermocouple reading. The reference temperature sensor may have poor accuracy (±1 K is not uncommon, and for simpler instruments, it may not even be specified). Furthermore, there may be poor thermal contact between the reference junction and the reference sensor, resulting in a temperature gradient between the two. This is generally the most significant error and typically has the most extensive variation. In data loggers with multiple thermocouple inputs, the thermocouples are often connected to ordinary copper screw terminals on the instrument circuit board, so the reference junction is actually in the screw terminal, while the reference junction sensor may be placed somewhere nearby on the circuit board (Figure 1).

Heat generation in the instrument may cause temperature gradients in the circuit board and temperature differences between the reference sensor and screw terminals and between screw terminals for the different input channels. This may cause additional errors, usually of the order of some tenths of a degree. Still, errors up to 2 K have been reported for rack-mounted systems due to temperature variations in the instrument cabinet [7]. Thus, a thermocouple instrument is affected by the environmental temperature. The device should be kept powered on in the measuring environment, long enough to reach a stable temperature before measurements. It is also essential to prevent the instrument from environmental thermal impact, e.g., from direct sun radiation. Note that errors due to thermal effects in the reference junction usually cannot be removed through calibration. Besides errors in the reference junction, there are also instrument errors, such as drift in offset and gain, but these errors are mostly negligible for modern instruments.

*Method errors* are situation-dependent systematic errors of physical character and refer to any differences between the temperature of the thermocouple tip and the temperature of the physical object we intend to measure on. Poor thermal contact between the sensor and object and heat transport through the thermocouple wires to or from the measuring junction are common causes of these types of errors. For temperature measurements in low-velocity airflows, radiation impact is usually the dominating source for this kind of errors [14].

## 4. Error Considerations Specific to Indoor Temperature Measurements

The influences from the different error sources mentioned above varies with the temperature range. It was found that, for indoor temperature measurements, inhomogeneity effects may almost always be ignored, and errors due to tolerance in the voltage–temperature curve of the thermocouple have a minor impact, while errors in the reference temperature compensation may be of paramount importance.

### 4.1. Measurement of Inhomogeneity Effects for a Thermocouple Type T in the Room Temperature Range

**Figure 2:**
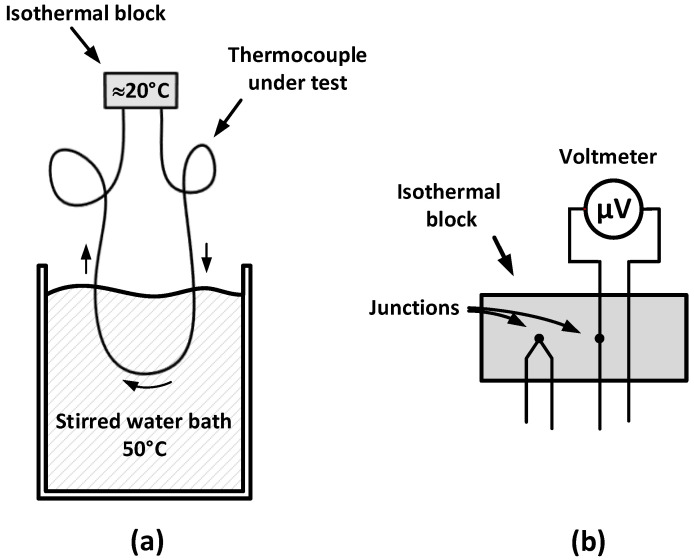
Inhomogeneity test. (**a**) Water bath. (**b**) Connection of the junctions. Both junctions were kept at the same temperature utilizing an isothermal aluminum block.

Measurements of thermocouple errors due to gradients are presented in many papers but only for comparably high gradients of several hundred degrees, e.g., [13,15]. Therefore, it was decided to investigate influence from temperature gradients in an environment typical for indoor climate applications, with gradients of a few decades of degrees. This investigation was performed by keeping both thermocouple junctions at the same temperature (room temperature) and applying a temperature gradient by dipping a loop of the wire in a hot water bath that holds a temperature of 50 °C (Figure 2).

Thus, two equal gradients of approximately 30 K were created about 0.1 m apart on the wire at the points where the wire passes the water–air barrier. The loop was moved along the wire in 0.2 m steps while measuring the thermocouple voltage with a voltmeter. Four different 3-m lengths of premium grade thermocouple wire Type T of diameter 0.13 mm and 0.2 mm of two different brands were tested. Here we used OMEGA TFCP-005 (Omega Engineering Inc. 800 Connecticut Ave. Suite 5N01, Norwalk, CT, USA) and Labfacility XE-2342 (Labfacility Limited, Eden Place, Sheffield, UK). No measurable output voltage was noticed for any of the tested wires. The noise floor of the measurement setup was in the range ±0.2 µV, corresponding to temperatures of ±0.005 K.

In indoor climate situations, where the measuring and reference junctions often reside in the same room, the gradients along the thermocouple are usually no more than a few degrees. Thus, even though a ±0.5 K offset is given in the IEC standard, the conclusion must be that errors due to inhomogeneities are negligible for indoor temperatures. This implies that for a premium grade Type T thermocouple used for room temperature measurements (and with the reference junction at room temperature), we may safely assume a pure gain error of maximum ±0.4 % of the temperature difference between the measuring and reference junctions and minimal additional offset.

### 4.2. Thermocouple Errors vs. Reference Junction Errors at Different Temperature Ranges

An error in the reference temperature compensation may cause a much larger percentual measurement error in room temperature measurements than in higher temperatures. Consider the following examples where we make temperature measurements with a thermocouple Type T of premium grade and an instrument where the accuracy for the reference junction is given to ±1 K (a typical value for a medium-priced instrument).

Suppose we measure the temperature in an oven that holds 300 °C. The reference junction is at room temperature 20 °C, and the sensor error may be estimated at 0.4% of (300–20) K = 1.1 K, which gives a total error of 2.1 K. Thus, about 50% of the total error is due to the reference junction error.

Compare this to an indoor climate situation where the difference between the temperatures at the measuring and the reference junctions is small, so that most of the output signal stems from the reference junction, and the thermocouple only adds a small correction to the reference temperature. Suppose we measure an indoor air temperature of 25 °C with the reference junction at 20 °C. According to the discussion in the previous section, we assume virtually no offset error and, thus, we estimate the sensor error to be 0.4% of (25–20) K = 0.02 K and, adding the reference junction error of 1 K, the total error will be 1.02 K. Here, the reference junction error constitutes 98% of the total error!

Obviously, a lower reference junction error would be of utmost benefit for room temperature measurements. The easiest way to decrease errors in the reference junction compensation is to disconnect the internal reference compensation in the instrument and instead use an external reference compensation device, which grants for higher accuracy. Some instrument manufacturers supply external reference compensation as an option for their instruments, but such a device can also be assembled by the user. There are instruments with tighter specifications of the reference junction for special applications, but they tend to be very expensive, especially for multi-point measurements.

## 5. Comparison of Accuracy between Measurements Using an External Reference Junction vs. Measurements with Internal Reference Junction Compensation—A Practical Case

### 5.1. An External Reference Junction Device

**Figure 3:**
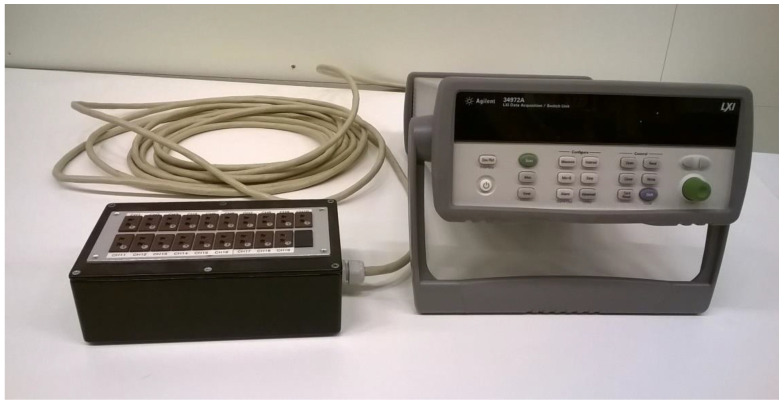
The data logger along with the reference junction box. The thermocouples are connected to the miniature thermocouple sockets at the top of the box. The cable from the box connects to the data logger DC voltage input channels.

A high-precision reference junction device can easily be assembled in the laboratory. It may consist of an aluminum block to which the thermocouple reference junction is attached together with a precision temperature sensor. Figure 3 shows an example of a reference junction device for 19 thermocouples intended for use together with a Keysight34970A/34972A data logger. (Keysight Technologies, 1400 Fountaingrove Parkway, Santa Rosa, CA, USA).

**Figure 4:**
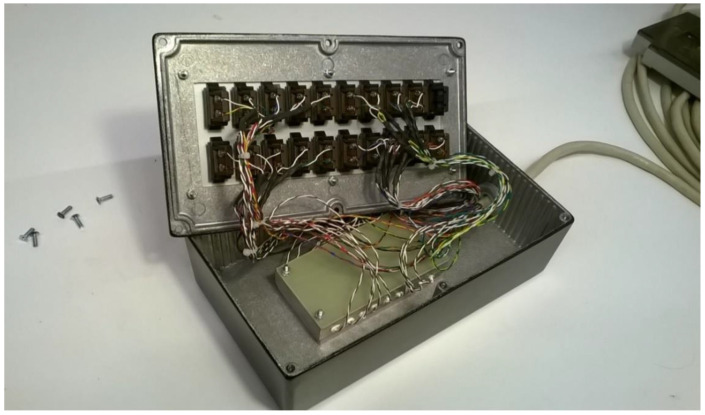
Inside the reference junction box. The aluminum block at the bottom of the box accommodates the reference junctions.

Thermocouples are connected via thermocouple connectors. To achieve a good thermal contact, the reference junctions for the thermocouples are mounted with the aid of heat conductive glue in holes drilled in the aluminum block. The reference temperature sensor is also mounted in the block. Here we used a precision thermistor of type PR103J2 with an accuracy of ±0.05 K (Littelfuse Inc. 8755 West Higgins Road Suite 500, Chicago, IL, USA). The aluminum block, which measures approximately 80 × 40 × 15 mm^3^, is mounted thermally insulated from the outer box by a polystyrene foam slab to minimize temperature influence from the box and its surroundings. See Figure 4.

The signals from the thermocouples and the thermistor are connected to the Keysight logger via an input connector module that plugs in at the rear of the logger. Each module contains 20 analog channels, of which 19 input channels are configured for DC voltage measurements from the thermocouples, while the remaining input connects to the precision thermistor. From the reference junction temperature, and the measured voltage from the respective thermocouple, the corrected temperatures are calculated using the standard 7th order polynomials for thermocouples Type T.

An additional practical advantage of the external reference junction device is that it allows for shorter thermocouple wires. The Keysight logger can accommodate three input connector modules with 20 analog channels in each. Thus, three cold junction boxes may be connected and placed at different positions. This avoids messing up the measurement space with long thermocouple wires.

### 5.2. Measured Accuracy Using Internal vs. External Reference Junction

In order to evaluate the accuracy, the temperature in a controlled water bath was measured. As temperature reference, a platinum thermometer calibrated to an accuracy of ±0.005 K was used. Measurements were performed with four thermocouples at four temperatures in the room temperature range. The thermocouples were Labfacility type XE-2322 premium grade of diameter 0.2 mm cut from the same spool.

The results from the measurements using the external reference junction box are displayed in Table 1. Excellent conformity in the measured values is revealed with a spread of no more than 0.01 K between the individual thermocouples. This is the same as the resolution of these measurements and indicates the excellent performance of the reference junction compensation. We note an equal difference to the reference thermometer of 0.01 to 0.05 K for the thermocouples in the measured temperature range, which must be attributed to nonlinearity in the thermocouples. The thermocouples show a higher temperature (≈0.02 K) than the reference thermometer for all the measured values. To what extent this is caused by thermocouple errors or by tolerance in the thermistor, measuring the reference junction temperature cannot be assessed from these measurements. With the external reference junction and the thermocouples used here, the maximum difference between the measured temperatures and the Reference thermometer was 0.05 K. This is less than the specified accuracy for the reference junction thermistor and thus the contribution from the thermocouples to the error budget is not measurable in this test. A quick check was made with thermocouples of other brands, Omega and TC Ltd. (TC Ltd., P.O. Box 130, Uxbridge, UK), which showed equally low measuring errors. This was expected, as the contribution from the thermocouple on the total error is small.

For comparison, measurements were also made using the same four thermocouples (XE-2322) and the same data logger but with the thermocouples connected directly to the data logger input module using the internal reference junction compensation (Table 2).

The logger had been switched on for 12 h before measurements, and the room temperature was stable. The results from these measurements expose an absolute error of up to 0.8 K and with opposite signs compared to the preceding measurements. The connections of the thermocouples were made such that the four thermocouples were spread out over the 20-channel screw terminal block in the input connector module with TC1 and TC4 at the ends of the connector. The measurement result clearly reveals a temperature gradient along the terminal block of up to 0.4 K with higher values at the ends of the screw terminal block. The linearity error noticed in the previous measurements also appear here as the same thermocouples are used in both measures. Interestingly, all thermocouples now show lower temperatures (on average 0.5 K) than the reference thermometer. Note that, as the same thermocouples were used in both measurements, errors in the thermocouples do not affect the results from this test. We must conclude that the differences in measured temperatures presented in Table 1 and Table 2 stem from inaccuracy in the internal reference junction temperature. However, the errors encountered are within the manufacturer’s specification for the Keysight data logger, where a maximum error of 1.0 K is stated for thermocouples of Type T in the range −100 to 400 °C [16].

## 6. Thermocouples in Indoor Air Temperature Measurements

### 6.1. Influence of Radiation

Measurements of air temperatures indoors need special attention. Air temperature measurements in a quiescent indoor environment are prone to significant errors due to the low thermal conductivity of air. The reading of a temperature sensor may differ from the actual air temperature because the sensor exchanges radiation with surrounding surfaces such as room walls and windows. Unless the temperature of the surfaces surrounding the sensor equals the air temperature, the sensor will attain a temperature between the temperatures of the air and the surroundings. At the usually relatively low indoor air velocities, the convective heat exchange between sensor and air is low enough for radiation heat exchange to be significant. The measurement error may be estimated through heat balance calculations for the sensor. However, the influential factors behind the calculations are difficult to assess in practice. Here we use an empirical method for quantifying the radiation sensitivity for a temperature sensor.

### 6.2. The Radiation Sensitivity Factor (RSF)

The temperature reading of a temperature sensor in air is a result of the energy balance at the sensor. For indoor air temperature measurements, heat transfer via convection at the sensor boundary layer and by radiation exchange between the sensor and surrounding surfaces are prevailing. The measurement error at steady-state conditions thus depends on the reflective properties of the involved surfaces, the size, and shape of the sensor, as well as the flow field surrounding it. For a temperature sensor immersed in air and being in thermal balance, the heat transfer due to radiation will be balanced by convective heat transfer so that:

(1) $$q_{rad}=q_{conv}$$

In an indoor temperature situation, both radiative and convective heat transfer may be considered to wary linearly with temperature, e.g., [14,17], and the steady-state energy balance at the sensor may be written as:

(2) $$h_{conv}\left(T_{sens}-T_{air}\right)=h_{rad}\left(T_{wall}-T_{sens}\right)$$

where $h_{conv}$ and $h_{rad}$ are the heat transfer coefficients for convection and radiation, respectively. $T_{air}\;$ is the air temperature, and the surface temperatures of the sensor and surrounding walls are denoted as $T_{sens}\;\mathrm{and}\;T_{wall}$, respectively.

Equation (2) is of limited use in a practical measuring situation due to difficulties estimating proper values for the heat transfer coefficients. Furthermore, the lead wires close to the sensors may take part in the heat transfer process, and an underlying problem is the dependency on details of the involved fluid mechanics as the convection heat transfer is dependent on the characteristics of the airflow field around the sensor, including flow direction and turbulence intensity. Lundström and Mattsson [14] have shown that for practical measurements, the influence from radiation on a temperature sensor may be expressed through the *Radiation Sensitivity Factor*, defined as $\;RSF=h_{rad}/h_{conv}$ and Equation (2) may be written as:

(3) $$\left(T_{sens}-T_{air}\right)=RSF\left(T_{wall}-T_{sens}\right)\;$$

*RSF* indicates the relationship between radiation and convection heat transfer at a sensor, quantifying to what extent the temperatures of surrounding surfaces affect the measured temperature. *RSF*, which must be established by measurements, is a function of emissivity, size, and shape of the sensor as well as on the flow field surrounding it. For example, a small sensor size and low emissivity make a low radiation sensitivity.

### 6.3. A Simple Method for Lowering the Radiation Sensitivity of a Thermocouple

**Figure 5:**
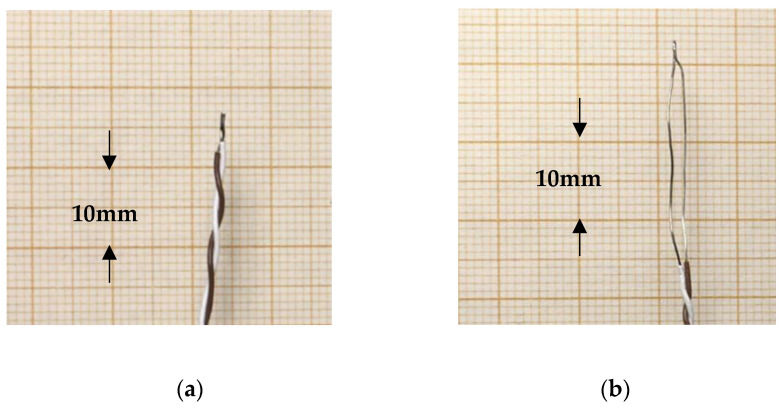
Thermocouples with two different measuring junctions. (**a**) Normal style. (**b**) Insulation off close to the tip.

As parts of the thermocouple wires close to the measuring junction take part in the measurements, stripping the insulation at the outermost part of the thermocouple leads and thereby exposing a metal surface is a simple means for decreasing the emissivity of the sensor (Figure 5).

**Figure 6:**
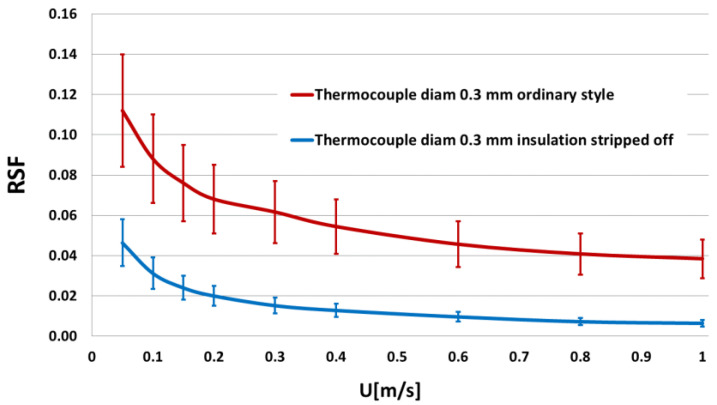
Radiation sensitivity factor for 0.3 mm thermocouple of ordinary style (red curve) and with stripped leads (blue curve) for different air velocities. The bars show the variation in data related to airflow direction: low bar ends = flow perpendicular to the sensor, high bar ends = flow along with the sensor. (Data from [14]).

Stripping a few centimeters of the insulation close to the tip will lower the RSF-value by more than 60% compared to a thermocouple insulated out to the junction. Figure 6 shows measured RSF-values for a 0.3 mm diameter thermocouple for the ordinary style and with stripped insulation.

Knowing the approximate mean radiant temperature and air velocity around a temperature sensor, the data in Figure 6 provides for calculating the errors caused by radiation for that sensor. The mean radiant temperature may be assessed from approximate temperatures and view factors of surrounding surfaces.

### 6.4. Sensitivity to the Airflow Direction

**Figure 7:**
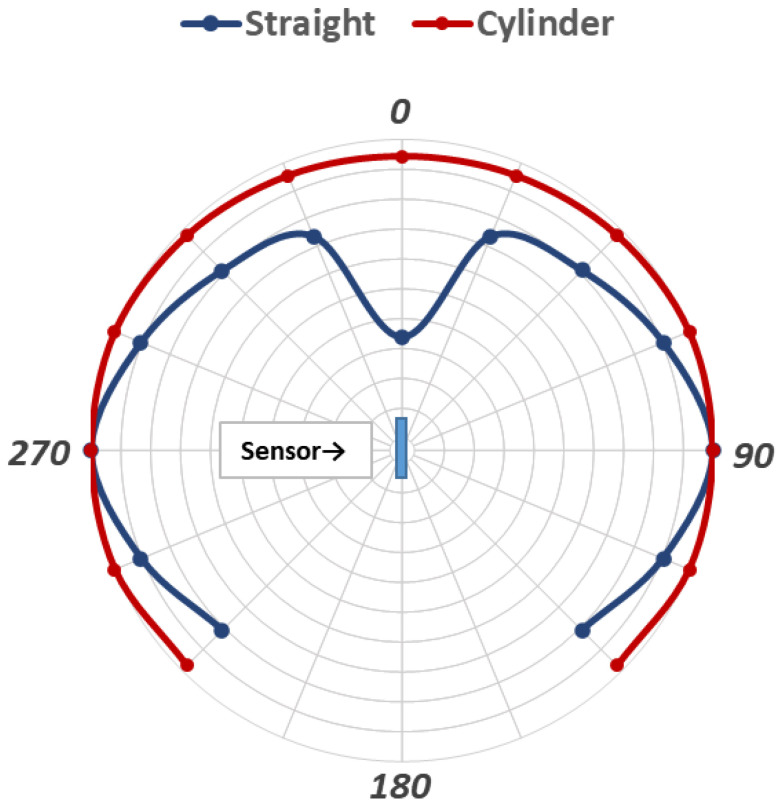
Relative heat convection for different airflow directions for straight (blue) and coiled (red) sensors. Zero degrees = flow head-on.

From Figure 6, we can see that the radiation sensitivity is dependent on the airflow direction relative to the sensor. This is due to variations in the airflow boundary layer and, thus, in the convection heat transfer at the sensor surface. Both for stripped and unstripped thermocouples, parts of the leads close to the measuring junction take part in the heat transfer, leading to that, a thermocouple sensor actually shows an elongated shape, more like a cylinder than a sphere. Thus, for a straight thermocouple, the convection heat transfer is dependent on the sensor’s orientation in the airflow. The convective heat transfer coefficient shows a maximum when the sensor axis is perpendicular to the flow and a minimum for flow along the sensor axis. The curves in Figure 6 shows mean values with the spread between the minimum and maximum at 0 and 90 degrees indicated by the error bars. The measured relative heat convection from flow of different directions for a straight thermocouple with stripped leads is indicated in the blue curve in Figure 7 with the head-on flow at zero degrees.

**Figure 8:**
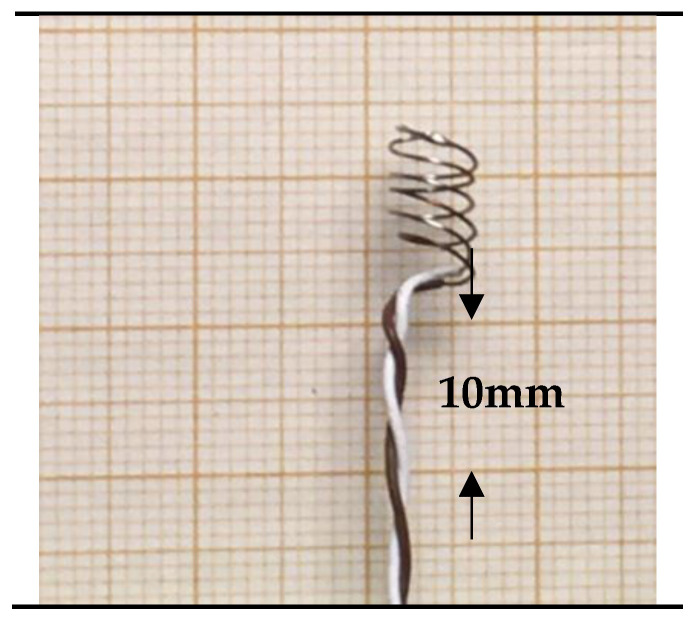
Stripped and coiled.

The flow direction may not be easily identified for low velocities indoors, and a more uniform convection heat transfer would be beneficial. This can be achieved by changing the shape of the sensor. For example, wrapping the stripped part of the thermocouple leads in the form of a coil, as shown in Figure 8, considerably decreases direction sensitivity. Measured values are shown in the red curve in Figure 7. Note that the curves in Figure 7 show relative sensitivities related to the 90-degree values.

## 7. Conclusions

For temperature measurements in the indoor temperature range, thermocouples show higher accuracy than as stated in Standards and commonly expressed in the literature. The weak point is often the reference junction compensation, which in most commercial thermocouple instruments suffer from bad accuracy. This error cannot be removed by individual calibration of the thermocouples. However, with an external reference compensation device, such as the one described in Section 5, measurements accuracies of ±0.05 K may be reached with a thermocouple Type T without foregoing calibration.

It may be worth mentioning that since this investigation was conducted, seven “cold junction boxes” have been manufactured and are now frequently used for temperature measurements in indoor climate research at The University of Gävle. Thermocouples of different brands are used with equally good performance.

Radiation impact on temperature sensors was also investigated. It was found that radiation impact from surrounding surfaces might generate significant errors for air temperature measurements in an indoor environment. The order of magnitude of errors due to radiation influence may be estimated from Figure 6. For example, suppose a thermocouple of 0.3 mm diameter with a soldered tip is used for air temperature measurements in a low-velocity environment, such as the occupied zone indoors, and the temperature difference between surrounding surfaces and the sensor temperature is 5 K. In that case, measurement errors on the order of 1 K may occur. The radiation impact may be reduced by up to 60% by stripping the insulation at the outermost part of the thermocouple leads, thereby exposing the wires’ metal surface close to the measuring junction.

## Figures and Tables

**Table 1:** Measured temperatures using an external reference junction box. Numbers in parenthesis show the difference to the reference thermometer.

Reference	Measured Temperatures			
Thermometer (°C)	Using External Reference Junction Box (°C)			
	TC1	TC2	TC3	TC4
8.65	8.69 (+0.04)	8.69 (+0.04)	8.69 (+0.04)	8.70 (+0.05)
18.83	18.86 (+0.03)	18.85 (+0.02)	18.85 (+0.02)	18.85 (+0.02)
28.81	28.83 (+0.02)	28.83 (+0.02)	28.83 (+0.02)	28.82 (+0.01)
38.80	38.81 (+0.01)	38.81 (+0.01)	38.81 (+0.01)	38.81 (+0.01)

**Table 2:** Measured temperatures using internal reference junction. Numbers in parenthesis the difference to the reference thermometer.

Reference	Measured Temperatures			
Thermometer (°C)	Using Keysight 34970A Internal Reference Junction (°C)			
	TC1	TC2	TC3	TC4
8.65	8.26 (−0.39)	8.44 (−0.21)	8.31 (−0.34)	7.85 (−0.80)
18.83	18.48 (−0.36)	18.59 (−0.25)	18.45 (−0.39)	17.98 (−0.86)
28.81	28.37 (−0.44)	28.54 (−0.27)	28.45 (−0.36)	27.99 (−0.82)
38.80	38.36 (−0.44)	38.54 (−0.26)	38.45 (−0.35)	38.00 (−0.80)
